# Real-World Effectiveness and Safety of Daridorexant in Japanese Patients With Insomnia: A Multicenter Observational Study Evaluating Four-Week Changes in Athens Insomnia Scale Among 54 Participants

**DOI:** 10.7759/cureus.97174

**Published:** 2025-11-18

**Authors:** Soichi Kono, Tasuku Nishiyama, Saki Nakano, Yoshikazu Ishimoto, Sho Horikoshi

**Affiliations:** 1 Psychiatry, Cosmos Street Psychosomatic Clinic, Fukushima, JPN; 2 Psychiatry, Hiiragi Kokoro Clinic, Fukuoka, JPN; 3 Data Science, Shionogi and Co. Ltd., Osaka, JPN; 4 Medical Affairs, Shionogi and Co. Ltd., Tokyo, JPN; 5 Psychiatry, Horikoshi Psychosomatic Clinic, Fukushima, JPN

**Keywords:** athens insomnia scale, daridorexant, dual orexin receptor antagonist, insomnia, psychiatric disorder

## Abstract

Background

Daridorexant, a dual orexin receptor antagonist (DORA), was recently approved in Japan for the treatment of insomnia. The drug has been shown to improve daytime functioning and has characteristics such as a lower risk of residual effects and rebound insomnia. Nevertheless, important exclusion criteria in clinical trials included patients with comorbid psychiatric disorders and those showing incomplete or insufficient responses to existing therapies. Therefore, this observational study assessed the effectiveness and safety of daridorexant in real-world settings for Japanese participants with insomnia beyond those evaluated in previous clinical trials.

Methods

The medical records of participants treated with daridorexant at three medical sites in Japan between December 2024 and May 2025 were reviewed. Participants were required to provide voluntary consent, to be 18 years or older, to be diagnosed with insomnia, and to have started daridorexant 50 mg treatment. They also needed an Athens Insomnia Scale (AIS) total score of 6 or higher. Participants with comorbid psychiatric disorders excluded from past clinical trials were also included in this study. Participants who did not comply with the dosage and administration instructions outlined in the package insert for daridorexant were excluded. The primary endpoint was the mean change in the AIS total score from baseline to week 4 of daridorexant treatment. The other endpoints included the percentage of participants achieving an AIS total score of 5 or lower after treatment and adverse events (AEs).

Results

Of 56 eligible participants for this study, 55 were included in the efficacy and safety analysis. Among the 55 participants, 36 (65.5%) were women, and the mean age was 43.4 years. Twenty-eight (50.9%) participants had moderate symptoms according to the AIS classification (mean AIS total score: 12.2), and all participants had comorbid psychiatric disorders. Twenty-eight (50.9%) were hypnotic-naïve, while the remaining 27 (49.1%) were taking some form of hypnotic medication, which included benzodiazepines (BZDs) (23.6%), DORAs (21.8%), and non-BZDs (18.2%).

One participant in whom daridorexant treatment was discontinued prior to the AIS evaluation in week 4 was excluded from the AIS evaluation. The mean change in AIS total score from baseline was -6.0 (95% confidence interval (CI): -7.2, -4.8, p<0.001), and 46.3% (25/54) of the participants achieved an AIS total score of ≤5 after four weeks of daridorexant treatment. Improvements were also observed across all eight AIS items, including daytime function (AIS-7). Significant improvements in AIS scores were observed in the subgroup of participants with depression, anxiety disorder, and schizophrenia. Daridorexant improved the AIS total score regardless of prior hypnotic medications. Eight participants experienced nonserious treatment-emergent AEs (TEAEs).

Conclusion

This study provided valuable insights into the real-world characteristics of outpatients initiating daridorexant therapy and showed its clinical effectiveness for insomnia, even in participants with psychiatric disorders and those with prior treatment failure. Improvements in daily function and a favorable safety profile were also observed in this participant population. These findings should be interpreted with caution, given the study's limitations, including a small sample size (n=54), short follow-up (four weeks), single-arm, and pre-post observational design.

## Introduction

In a nationwide epidemiological study conducted in Japan among adults aged ≥20 years, the prevalence of insomnia was reported to be 12.2% in men and 14.6% in women [1]. Meta-analyses, along with data from national health interview surveys and cohort studies, have identified insomnia as a risk factor for psychiatric disorders including depression, anxiety disorder, and adjustment disorder [2-4], and an umbrella review of meta-analyses of cohort studies demonstrated that insomnia is associated with an increased relative risk of atrial fibrillation, myocardial infarction, and stroke [5]. In addition, a cohort study conducted within an Australian community highlighted the need for early detection and management of insomnia, due to its impact on workplace productivity [6].

Optimal insomnia treatment should be individualized based on the type of symptoms, the patient's age and individual situation, the presence of complications, and potential drug interactions. In this context, clinicians must be aware of the risks related to long-term use of hypnotics associated with irrational polypharmacy [7] and adverse reactions associated with the prolonged use of benzodiazepines (BZDs) and/or non-BZDs (Z-drugs) [8]. The European Insomnia Guideline [9], as revised in 2023, recommends daridorexant for short-term insomnia treatment (≤ 4 weeks) when cognitive-behavioral therapy is ineffective. Furthermore, the guideline also recommends its use for periods of up to three months or longer in some cases, considering the advantages and disadvantages. In addition, the Japanese expert consensus recommends the use of a dual orexin receptor antagonist (DORA) as the first-line treatment for both sleep onset and maintenance in treatment-naïve individuals with insomnia [10]. As a result of these circumstances, recent prescription trends in Japan indicate a shift, with BZD prescriptions declining between 2013 and 2021, while DORA prescriptions steadily increasing [11].

Daridorexant, the third DORA introduced in Japan (December 2024), promotes sleep by antagonizing orexin receptors in the hypothalamus. Its short half-life (6.33 hours, on day 4 of a daily 50 mg dose in adults aged 20-50 years) may be involved in reducing the risk of residual effects, withdrawal, and rebound insomnia [12-14]. These clinical studies on daridorexant have demonstrated improvements not only in sleep duration but also in sleep quality and other patient-reported outcomes. However, the clinical trials conducted for its approval in Japan involved patients with primary insomnia, whereas patients with acute, unstable, or treatment-requiring psychiatric conditions were excluded [12,13]. Real-world clinical practice often involves patients with psychiatric disorders or those with inadequate responses to existing treatments. When this study was initiated, daridorexant had only recently been launched in Japan, and it remained uncertain whether the benefits observed in controlled clinical trials would translate to effectiveness and safety in real-world clinical practice, particularly in patients with comorbid psychiatric conditions. Therefore, the actual effectiveness and safety of daridorexant in these populations warrant further investigation.

Against this background, we conducted an observational study of daridorexant across multiple psychiatric outpatient clinics, where board-certified psychiatrists provide care to individuals primarily presenting with insomnia, frequently accompanied by mild to moderate psychiatric disorders.

This observational study aimed to evaluate the real-world effectiveness and safety of daridorexant in Japanese participants with insomnia. The primary objective was to assess changes in insomnia severity, as measured by the Athens Insomnia Scale (AIS) total score, over a four-week treatment period. Secondary objectives included evaluating the proportion of participants achieving remission (defined as an AIS score ≤ 5) and documenting the incidence and nature of adverse events (AEs). These objectives were designed to determine whether the clinical benefits of daridorexant observed in randomized controlled trials would be replicated in routine psychiatric practice, particularly among patients with comorbid psychiatric conditions who were typically excluded from clinical studies.

## Materials and methods

Participants and procedures

This non-interventional, observational study enrolled participants who visited one of three psychiatric clinics (Cosmos Street Psychosomatic Clinic, Horikoshi Psychosomatic Clinic, or Hiiragi Kokoro Clinic) between December 2024 and May 2025. After their visit, eligible participants who were subsequently diagnosed with insomnia according to the Diagnostic and Statistical Manual of Mental Disorders, Fifth Edition (DSM-5), were initiated on daridorexant 50 mg once daily. Informed consent was obtained verbally or in writing, using a document approved by the ethics review committee, from all participants who started daridorexant as part of routine care. We employed consecutive sampling during the study period to minimize selection bias. All patients who visited the participating clinics during the enrollment period, met the inclusion and exclusion criteria, and provided informed consent were invited to participate in the study. No random selection or preferential enrollment was conducted, ensuring that eligible patients were enrolled consecutively without investigator selection bias. The existing data in their medical records were then retrieved and analyzed. Although sleep diaries were utilized in clinical trials to evaluate the efficacy of daridorexant, we utilized the Japanese version of the Athens Insomnia Scale (AIS) as our primary sleep assessment tool, which we use in our daily clinical practice to diagnose insomnia and its related symptoms [15]. The use of the Japanese version of AIS in this study was permitted by the original authors. AIS is an eight-item self-report questionnaire that assesses insomnia symptoms during the last month. The Likert-type scoring scale ranges from 0 (indicating the item in question has not been a problem) to 3 (indicating more acute sleep difficulties) per item, with a total score ranging from 0 to 24. The severity of insomnia is classified as follows: 0-5, absence of insomnia; 6-9, mild insomnia; 10-15, moderate insomnia; and 16-24, severe insomnia. The scale has been validated against the International Classification of Diseases, 10th Revision (ICD-10) diagnostic criteria, with a total score of ≥6 correctly identifying the presence of "nonorganic insomnia" in 90% of study subjects [16].

The inclusion criteria were as follows: voluntary informed consent to participate in the study; age of 18 years or older; diagnosis of insomnia at the study site and initial treatment with daridorexant 50 mg between December 2024 and May 2025; available AIS score on the day of treatment initiation (baseline) and at week 4 (28±7 days after treatment initiation); and AIS total score of 6 or higher on day 1 of daridorexant treatment. The exclusion criteria were as follows: failure to meet the indications or presence of contraindications for daridorexant; failure to follow the dosage and administration instructions in the package insert for daridorexant; and history of suicidal ideation, suicide attempts, self-harm, or drug abuse.

The primary endpoint was the change in the AIS total score from baseline to week 4 of daridorexant treatment. The secondary endpoint was the frequency of adverse events (AEs). Exploratory endpoints were changes in individual AIS item scores, the proportion of participants achieving an AIS total score of ≤5 at week 4, and subgroup analyses by psychiatric disorder and hypnotic medication status (add-on or switched).

Participants who had not used any hypnotic medication for at least 28 days prior to baseline were defined as hypnotic-naïve. Switching was defined as discontinuing other hypnotics before starting daridorexant, while add-on referred to the addition of daridorexant to ongoing hypnotic therapy.

Data were collected using an electronic data capture (EDC) system, including anonymized information on demographics, treatment status, concomitant medications, efficacy outcomes, adverse events, and reasons for discontinuation of daridorexant treatment.

Statistical analysis

As this was an exploratory observational study aimed at describing real-world effectiveness, no target sample size was set. For reference, based on the results of previous daridorexant clinical trials [13], we estimated that approximately 26 cases would be needed to detect a statistically significant difference in changes in AIS scores from baseline. Participants with violations of the informed consent process or serious deviations from study procedures were to be excluded from the safety analysis set. The full analysis set was considered identical to the safety analysis set. In addition, participants in whom daridorexant treatment was discontinued before the evaluation at week 4 were excluded from the efficacy assessment.

Descriptive statistics were used for continuous and categorical variables. The Shapiro-Wilk test was performed to assess the normality of the changes in AIS scores. Based on the results, changes in AIS scores were analyzed using paired t-tests when normality was not rejected, or Wilcoxon signed-rank tests when normality was rejected, with a two-sided significance level of 5%. Ninety-five percent confidence intervals (CIs) were calculated for the changes. Statistical analyses were performed using SAS 9.4® software (SAS Institute, Cary, NC).

The numbers and percentages of participants with AEs that occurred in the safety analysis set were tabulated. AEs were coded using MedDRA version 28.0.

Ethics

This study was conducted in accordance with the ethical principles stipulated in the Declaration of Helsinki (revised in 2024) and in compliance with the Ethical Guidelines for Human Life Science and Medical Research Involving Human Subjects (enforced on June 30, 2021, and partially amended), the Act on the Protection of Personal Information (including Act No. 57 of 2003 and amendments), related laws and regulations, related notifications, and this study protocol. The study protocol was approved by the Research Ethics Committee of the Non-Profit Organization MINS Institutional Review Board (approval number: 250212).

## Results

A total of 56 participants (19 male participants and 37 female participants) were enrolled in the study, with approximately two-thirds of the participants being women. One female participant met an exclusion criterion and was excluded from both the full analysis set and the safety analysis set. The participants' demographics and clinical characteristics are summarized in Table 1. All participants had at least one psychiatric comorbidity. The mean±standard deviation (SD) age was 43.4±15.4 years, with the majority (n=49) being under 65 years of age. Based on the AIS total score, the severity of insomnia was approximately distributed in a 1:2:1 ratio across the mild, moderate, and severe categories. Twenty-seven participants had received at least one medication for insomnia prior to initiating daridorexant.

**Table 1:** Participants' demographics and clinical characteristics A total of 56 participants were enrolled in the study. One participant met an exclusion criterion after daridorexant treatment was initiated and was excluded from the full analysis set. ^a^Breakdown of psychiatric disorders classified by the number of participants with each psychiatric disorder was as follows: depression (n=33), anxiety disorder (n=17), bipolar disorder (n=10), developmental disorder (n=7), schizophrenia (n=6), adjustment disorder (n=4), and others (n=6). AIS: Athens Insomnia Scale (maximum total score of 24 points), BZD: benzodiazepine, Z-drug: non-benzodiazepine hypnotics, FAS: full analysis set, SD: standard deviation

Variable	Result
Number of FAS, number	55
Age, mean±SD, years	43.4±15.4
<65 years, number (%)	49 (89.1)
≤65 years, number (%)	6 (10.9)
Sex	
Male, number (%)	19 (34.5)
Female, number (%)	36 (65.5)
Height, mean±SD, cm	161.1±9.7
Weight, mean±SD, kg	63.8±16.3
Duration of insomnia, mean±SD, years	3.1±5.1
Insomnia, AIS at baseline, mean±SD	12.7±4.0
6-9, number (%)	13 (23.6)
10-15, number (%)	28 (50.9)
≥16, number (%)	14 (25.5)
Prior treatment for insomnia	
No, number (%)	28 (50.9)
Yes, number (%)	27 (49.1)
Number of prior medications for insomnia	
One drug, number (%)	15 (27.3)
Two drugs, number (%)	11 (20.0)
Three or more drugs, number (%)	1 (1.8)
Prior medications for insomnia	
Lemborexant, number (%)	10 (18.2)
Suvorexant, number (%)	2 (3.6)
Z-drug, number (%)	10 (18.2)
BZD, number (%)	13 (23.6)
Melatonin preparation, number (%)	2 (3.6)
Others, number (%)	2 (3.6)
Psychiatric disorder treatment	
No, number (%)	7 (12.7)
Yes, number (%)	48 (87.3)
Concomitant psychiatric disorder^a^	
No, number (%)	0 (0.0)
Yes, number (%)	55 (100.0)
Concomitant other disease	
No, number (%)	29 (52.7)
Yes, number (%)	26 (47.3)

The most frequently prescribed insomnia medications prior to daridorexant were BZDs (n=13), DORAs (n=12), and Z-drugs (n=10). Among the DORA group, 10 participants had been treated with lemborexant and two with suvorexant. Comorbid psychiatric disorders included depression (n=33), anxiety disorder (n=17), bipolar disorder (n=10), developmental disorder (n=7), and schizophrenia (n=6).

In the AIS assessment, one participant who discontinued daridorexant prior to the week 4 evaluation was excluded, resulting in a final sample of 54 participants (Table 2). Table 3 presents changes in the AIS total score and item scores between baseline and week 4. The mean AIS total score decreased from 12.6±4.0 at baseline to 6.6±4.0 at week 4 (mean change = -6.0 (95% CI: -7.2, -4.8), p<0.001). The mean scores for all eight AIS items were lower at week 4 compared to baseline, with marked reductions observed for each item (p<0.001). These improvements encompassed not only core sleep-related items (AIS-1: sleep induction, AIS-2: awakening during the night, and AIS-3: final awakening) but also AIS-7, which evaluates daily functioning.

**Table 2:** AIS assessment in participants ^a^One participant who discontinued study treatment by week 4 was excluded from the analysis. ^b^Percentages based on the number of analysis sets (n=55). ^c^Percentages of AIS total score ≤ 5 based on the number of participants in which AIS were conducted. AIS: Athens Insomnia Scale, FAS: full analysis set

Variable	Result
Number of FAS, number	55
Participants who underwent AIS assessments^a,b^	
Baseline	54 (98.2)
Week 4	54 (98.2)
AIS total score ≤ 5^c^	
Baseline	0 (0.0)
Week 4	25 (46.3)

**Table 3:** Changes in the AIS total scores and each AIS questionnaire item in the participants AIS total scores and changes in scores of each AIS questionnaire item at baseline and at week 4 are presented. For the mean changes, the 95% CIs were calculated, and paired t-tests or Wilcoxon signed-rank tests were conducted depending on the results of the Shapiro-Wilk test for normality. Paired t-tests were used for AIS total scores, while Wilcoxon signed-rank tests were used for each of the eight AIS questionnaire items. AIS: Athens Insomnia Scale, CI: confidence interval, SD: standard deviation

Variable	Result
AIS total score	
Baseline, mean±SD	12.6±4.0
Week 4, mean±SD	6.6±4.0
Change from baseline, mean (95% CI)	-6.0 (-7.2, -4.8)
P-value for change	<0.001
t-statistic	-10.0
AIS-1 (sleep induction)	
Baseline, mean±SD	1.6±0.8
Week 4, mean±SD	0.6±0.7
Change from baseline, mean (95% CI)	-0.9 (-1.2, -0.7)
P-value for change	<0.001
S-statistic	-355.5
AIS-2 (awakening during the night)	
Baseline, mean±SD	1.6±0.7
Week 4, mean±SD	0.8±0.7
Change from baseline, mean (95% CI)	-0.8 (-1.0, -0.6)
P-value for change	<0.001
S-statistic	-269
AIS-3 (final awakening)	
Baseline, mean±SD	1.4±1.0
Week 4, mean±SD	0.8±0.8
Change from baseline, mean (95% CI)	-0.5 (-0.8, -0.2)
P-value for change	<0.001
S-statistic	-226
AIS-4 (total sleep duration)	
Baseline, mean±SD	1.5±0.9
Week 4, mean±SD	0.7±0.6
Change from baseline, mean (95% CI)	-0.8 (-1.1, -0.6)
P-value for change	<0.001
S-statistic	-313
AIS-5 (sleep quality)	
Baseline, mean±SD	1.8±0.7
Week 4, mean±SD	0.9±0.8
Change from baseline, mean (95% CI)	-0.9 (-1.1, -0.6)
P-value for change	<0.001
S-statistic	-327.5
AIS-6 (well-being)	
Baseline, mean±SD	1.6±0.8
Week 4, mean±SD	0.8±0.7
Change from baseline, mean (95% CI)	-0.8 (-1.0, -0.6)
P-value for change	<0.001
S-statistic	-348
AIS-7 (functioning capacity)	
Baseline, mean±SD	1.6±0.7
Week 4, mean±SD	0.8±0.9
Change from baseline, mean (95% CI)	-0.8 (-1.0, -0.5)
P-value for change	<0.001
S-statistic	-358.5
AIS-8 (sleepiness during the day)	
Baseline, mean±SD	1.5±0.7
Week 4, mean±SD	1.1±0.8
Change from baseline, mean (95% CI)	-0.5 (-0.7, -0.2)
P-value for change	<0.001
S-statistic	-190.5

**Figure 1:**
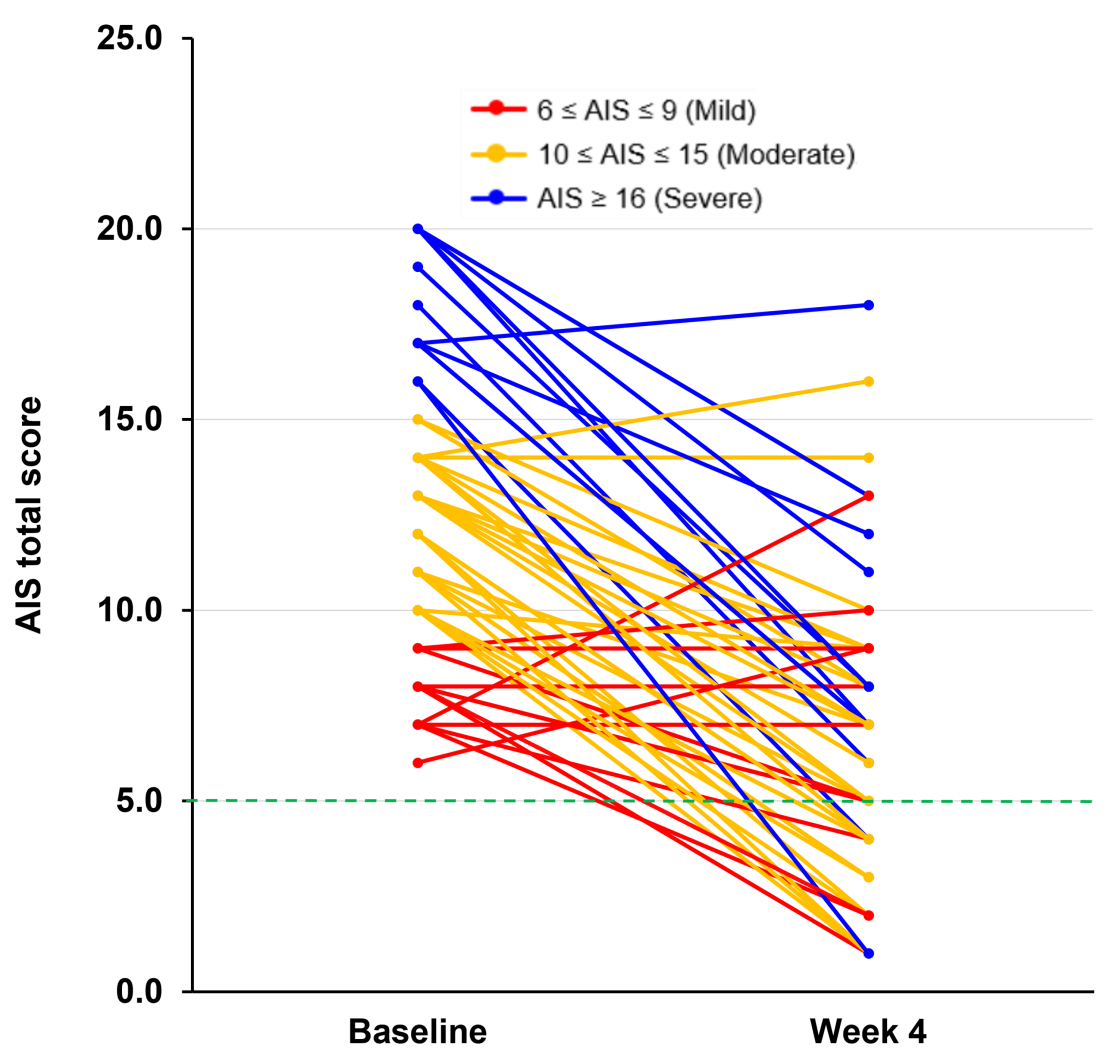
Individual changes in AIS total score from baseline to week 4 The individual changes in the AIS total score from baseline to week 4 for each participant are presented (n=54). Each change is indicated by color according to the insomnia severity based on AIS total scores at baseline: mild (6-9, shown as red), moderate (10-15, shown as orange), and severe (16 and above, shown as blue). Of the 54 participants, 25 (46.3%) showed improvement in their AIS total scores to 5 (green dashed line) or below by week 4. AIS: Athens Insomnia Scale

The AIS total score for each participant at baseline and week 4 is shown in Figure 1. Among the 54 participants, 45 demonstrated a reduction in the AIS total score from baseline to week 4, and this improvement was independent of baseline insomnia severity. Four participants showed no change in total score between baseline and week 4, whereas five participants exhibited an increase at week 4 compared to baseline. At week 4, 25 (46.3%) participants achieved an AIS total score of ≤5 (Table 2).

**Figure 2:**
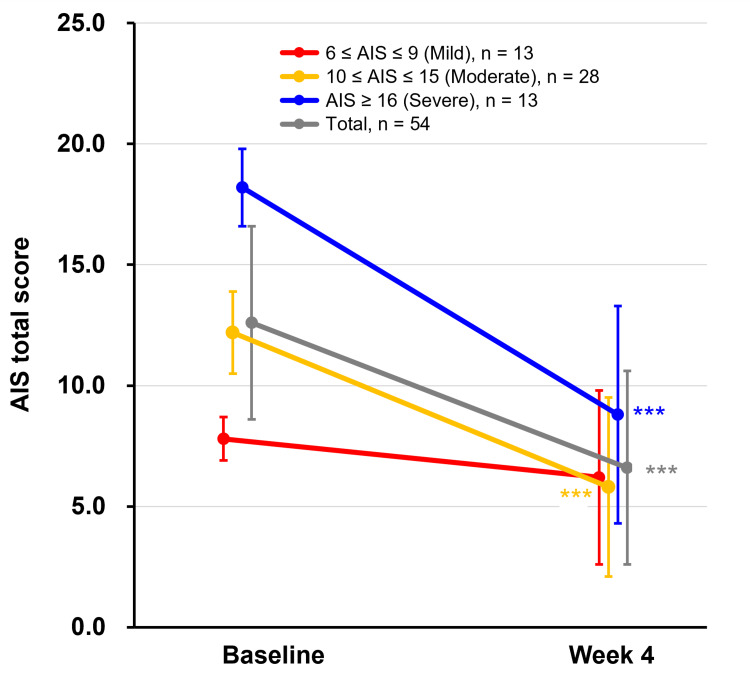
Changes in AIS total scores by insomnia severity The mean changes in AIS total scores from baseline to week 4 are shown by insomnia severity. The mean change in total participants is also represented in grey. Significant differences in the mean change from baseline were observed at week 4 in participants with moderate and severe insomnia, as well as in the total participant group (***p<0.001). Based on the results of the Shapiro-Wilk test for normality, paired t-tests were used for mild and severe insomnia groups, while Wilcoxon signed-rank tests were used for the moderate insomnia group. Data are presented as the mean±SD. AIS: Athens Insomnia Scale, SD: standard deviation

Changes in the AIS total score from baseline to week 4 by insomnia severity are illustrated in Figure 2. The mean AIS total score substantially improved in the severe group from 18.2±1.6 to 8.8±4.5, representing a mean change of -9.4 (95% CI: -11.9, -6.9, p<0.001). The moderate group also showed a decrease from 12.2±1.7 at baseline to 5.8±3.7 at week 4, with a mean change of -6.5 (95% CI: -7.6, -5.3, p<0.001). In contrast, the mild group exhibited only a slight reduction from 7.8±0.9 to 6.2±3.6 (mean change = -1.6 (95% CI: -3.9, 0.6), p=0.142). By four weeks of daridorexant treatment, AIS total scores of ≤5 were achieved by seven of 13 participants with mild insomnia, 16 of 28 with moderate insomnia, and two of 13 with severe insomnia.

**Figure 3:**
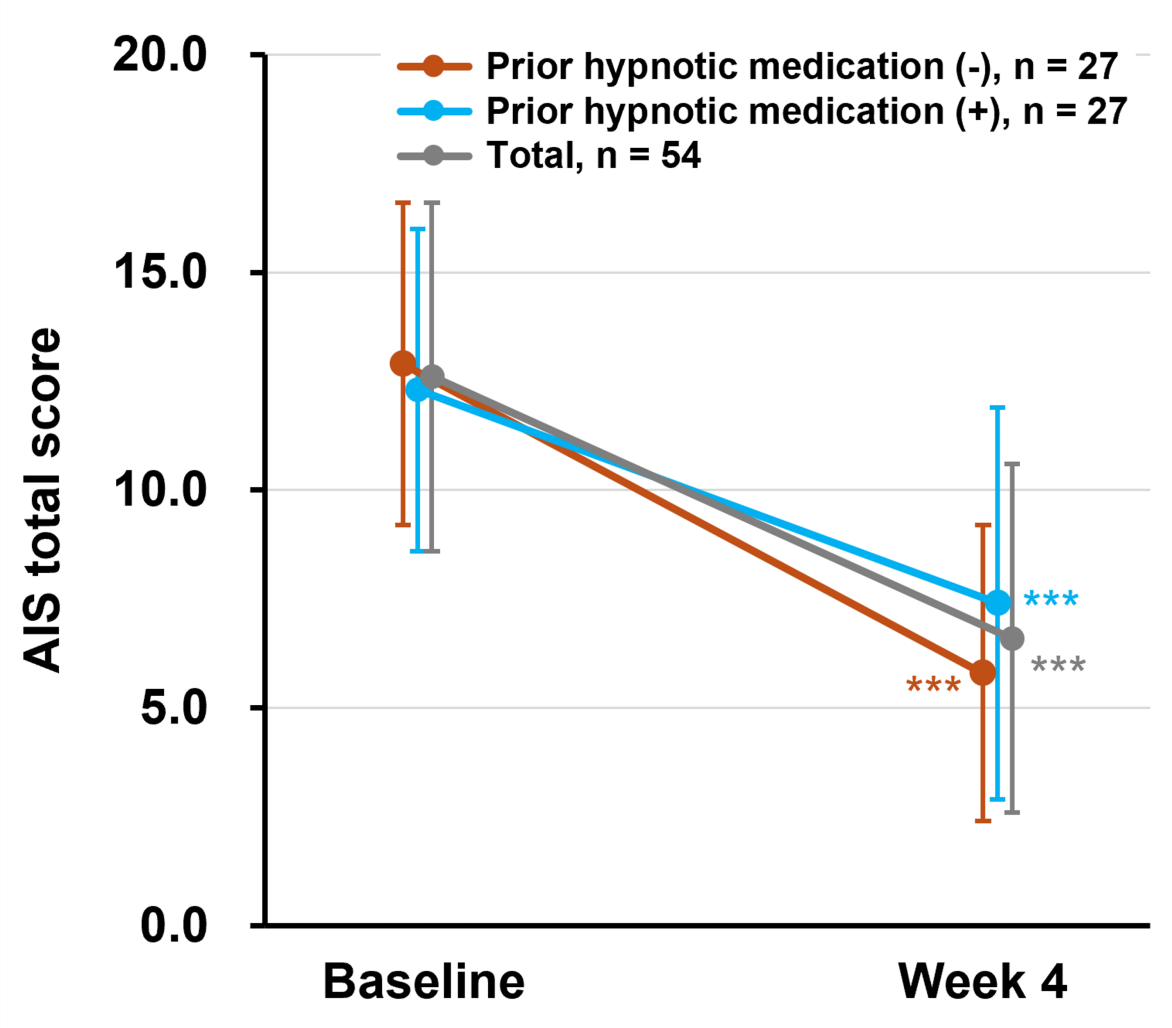
Changes in AIS total scores in participants with/without prior hypnotic medication The mean changes in AIS total scores from baseline to week 4 are shown based on whether prior hypnotic medication was used (+, shown as light blue) or not (-, shown as brown). The mean change in total participants is shown as grey. Significant differences in the mean change from baseline were observed at week 4 in participants with or without prior hypnotic medication, as well as in the total participant group (***p<0.001). Based on the results of the Shapiro-Wilk test for normality, paired t-tests were used for participants without prior hypnotic medication, while Wilcoxon signed-rank tests were used for those with prior hypnotic medication. Data are presented as the mean±SD. AIS: Athens Insomnia Scale, SD: standard deviation

Changes in AIS scores for participants with and without prior hypnotic medication use are shown in Figure 3. Among participants without prior treatment for insomnia (n=27), the mean AIS total score decreased from 12.9±3.7 at baseline to 5.8±3.4 at week 4, corresponding to a mean change of -7.1 (95% CI: -8.7, -5.5, p<0.001). Among participants with prior treatment (n=27), the mean AIS total score decreased from 12.3±4.3 to 7.4±4.5, with a mean change of -4.9 (95% CI: -6.7, -3.1, p<0.001).

**Figure 4:**
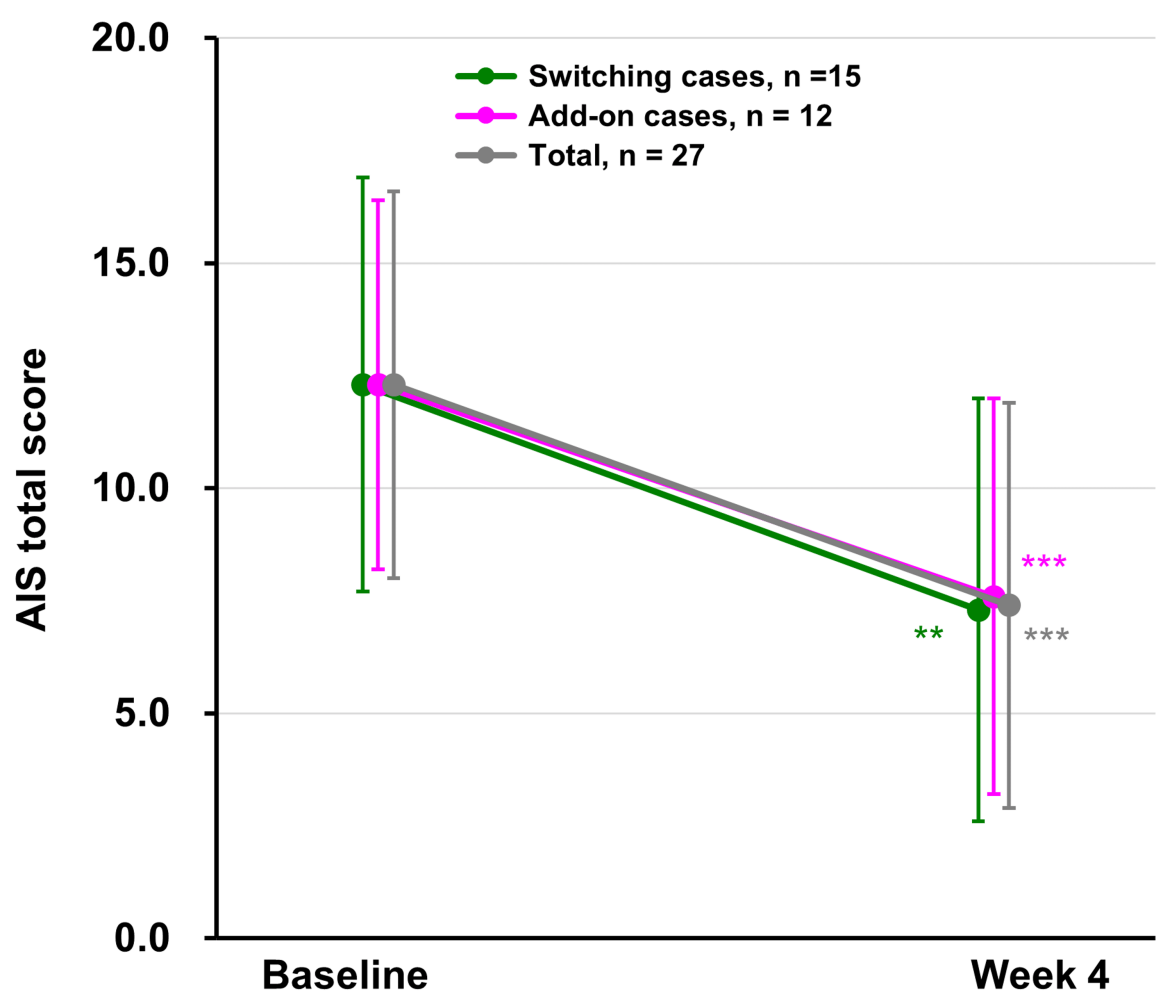
Changes in AIS total scores in participants who switched or had add-on cases The mean changes in AIS total scores from baseline to week 4 are shown in participants who switched (switching cases, shown as green) or had add-on (add-on cases, shown as pink) cases. The mean change for the combined total of both groups is shown as grey. Switching cases: where other hypnotics were discontinued and treatment with daridorexant was initiated. Add-on cases: where daridorexant was added to other hypnotics at baseline. Significant differences in the mean change from baseline were observed at week 4 in switching cases (**p<0.01) and add-on cases (***p<0.001), as well as in the combined total of both groups (***p<0.001). Based on the results of the Shapiro-Wilk test for normality, paired t-tests were used for add-on cases, while Wilcoxon signed-rank tests were used for switching cases. Data are shown as mean±SD. AIS: Athens Insomnia Scale, SD: standard deviation

Changes in mean AIS total scores among participants who switched to daridorexant (n=15) and those who received daridorexant as add-on therapy (n=12) are shown in Figure 4. The mean AIS total score decreased from 12.3±4.6 at baseline to 7.3±4.7 at week 4 among participants who switched treatment (mean change = -5.1 (95% CI: -8.0, -2.1), p=0.003) and decreased from 12.3±4.1 to 7.6±4.4 among those who received add-on treatment (mean change = -4.7 (95% CI: -6.9, -2.4), p<0.001).

**Figure 5:**
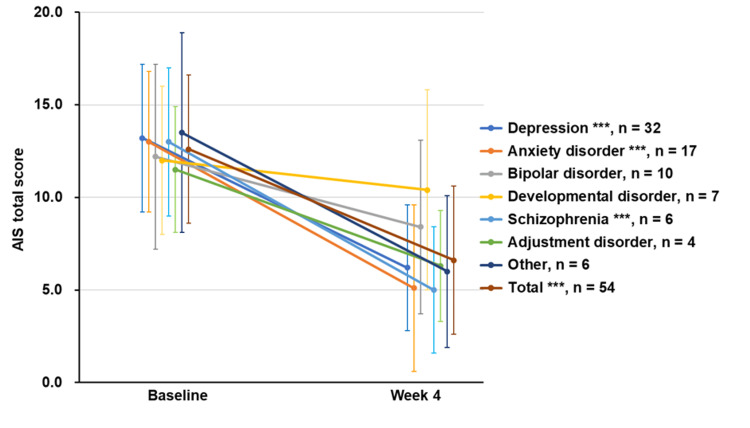
Changes in AIS total scores by type of psychiatric disorder The mean changes in AIS total scores from baseline to week 4 are shown in participants categorized by the type of psychiatric disorder. The mean change for total participants with any comorbid psychiatric disorder is shown in brown. Significant differences in the mean change from baseline were observed at week 4 in participants with depression, anxiety disorder, and schizophrenia, as well as in the total participants with any comorbid psychiatric disorder (***p<0.001). No significant differences were observed in participants with bipolar disorder (p=0.060), developmental disorder (p=0.236), adjustment disorder (p=0.069), or other psychiatric disorders (p=0.063). Based on the results of the Shapiro-Wilk test for normality, paired t-tests were used for all psychiatric disorder categories, except for other psychiatric disorders, where Wilcoxon signed-rank tests were used. Data are shown as mean±SD. AIS: Athens Insomnia Scale, SD: standard deviation

Mean changes in the AIS total score according to the type of psychiatric disorder are shown in Figure 5. Marked reductions in AIS total scores from baseline to week 4 were observed in participants with depression, anxiety disorder, and schizophrenia (p<0.001 for all), but not in those with bipolar disorder (p=0.060), developmental disorder (p=0.236), or adjustment disorder (p=0.069).

Table 4 summarizes the treatment-emergent adverse events (TEAEs). No participants discontinued daridorexant due to TEAEs during the assessment period. TEAEs were reported in eight participants. Malaise, reported in one participant, was the only TEAE considered related to daridorexant treatment. This participant continued daridorexant at a reduced dose of 25 mg once daily until study completion. All TEAEs were nonserious and resolved without requiring treatment discontinuation.

**Table 4:** Treatment-related adverse events Treatment-related adverse events were recorded for all participants from the start of daridorexant treatment to the last day of daridorexant treatment. Percentages were calculated based on the number of safety analysis sets. Participants with more than one TEAE were counted for each event, but were counted once for any TEAE. ^a^Malaise was the only case of treatment-related TEAE, and the dose reduction was implemented to manage the event. ^b^Adverse events were coded using MedDRA version 28.0. TEAE: treatment-emergent adverse event, SAS: safety analysis set

Variable	Result
Number of SAS, number	55
Discontinuation of the study, number (%)	0 (0.0)
TEAEs, number (%)	8 (14.5)
TEAEs leading to death, number (%)	0 (0.0)
TEAEs leading to discontinuation of study treatment, number (%)	0 (0.0)
Serious TEAEs, number (%)	0 (0.0)
Treatment-related TEAEs, number (%)	1 (1.8)^a^
Treatment-related TEAEs leading to death, number (%)	0 (0.0)
Treatment-related TEAEs leading to discontinuation of study treatment, number (%)	0 (0.0)
Treatment-related serious TEAEs, number (%)	0 (0.0)
TEAEs: classification and names^b^	
Gastrointestinal disorders, number (%)	4 (7.3)
Abdominal discomfort, number (%)	1 (1.8)
Chronic gastritis, number (%)	1 (1.8)
Gastritis, number (%)	1 (1.8)
Stomatitis, number (%)	1 (1.8)
Nervous system disorders, number (%)	2 (3.6)
Headache, number (%)	1 (1.8)
Somnolence, number (%)	1 (1.8)
General disorders and administration site conditions, number (%)	1 (1.8)
Malaise^a^, number (%)	1 (1.8)
Psychiatric disorders, number (%)	1 (1.8)
Schizophrenia, number (%)	1 (1.8)

## Discussion

Key findings

This observational study using medical records evaluated the effectiveness and safety of daridorexant in real-world settings among Japanese adults with insomnia. Daridorexant improved the AIS score in participants regardless of baseline insomnia severity or prior use of hypnotic medication. Following treatment initiation, both AIS total and individual item scores decreased, with nearly half of the participants achieving an AIS total score of ≤5 (indicative of adequate sleep) at week 4. Importantly, these improvements were observed in participants with psychiatric comorbidities, a population typically excluded from clinical trials. These findings suggest that daridorexant may serve as a promising and well-tolerated treatment option for insomnia, including those with psychiatric comorbidities and those with incomplete or insufficient responses to existing therapies.

AIS as a tool for measuring insomnia

The AIS is a self-assessment psychometric instrument consisting of eight items designed to quantify sleep difficulties by evaluating various dimensions of insomnia, including sleep quality, sleep quantity, and daytime functioning [17]. Owing to its high internal consistency, reliability, and validity, the AIS is widely regarded as an essential tool in both sleep research and clinical practice. The AIS has demonstrated strong correlation with the Insomnia Severity Index (ISI), a widely used tool for assessing insomnia severity (r=0.80, p<0.001), with established severity cutoff scores that align well with ISI-based classifications [18]. A meta-analysis comparing the diagnostic accuracy of insomnia screening tools demonstrated that the AIS and ISI exhibit comparable sensitivity (91% versus 88%) and specificity (87% versus 85%) for insomnia screening [19]. The participating institutions regularly incorporate the AIS into clinical care, and for this study, AIS scores were extracted from existing medical records for analysis. Objective sleep parameters from previous clinical trials, including reductions in sleep onset latency and wake time after sleep onset, as well as increased total sleep time [12], align with the present findings. Furthermore, daridorexant has been shown to suppress transitions from all sleep stages to wakefulness [20], further supporting its sleep-stabilizing effects.

Clinical impact of daridorexant on sleep and daytime function

Among the AIS items, improvement in daytime functioning (AIS-7) is particularly relevant to the therapeutic goals of hypnotic agents. The International Classification of Sleep Disorders, Third Edition, includes daytime dysfunction in the diagnostic criteria for chronic insomnia, emphasizing the growing importance of improving daytime function with hypnotics. In line with the results of clinical studies conducted in Japan and the USA [13,21], the present study revealed improvements in participants' self-reported daytime functioning based on the AIS-7 item (Table 3). A retrospective analysis in the USA similarly reported enhanced daytime functioning in patients who transitioned from other hypnotics to daridorexant [22], suggesting that this may be a distinguishing feature of daridorexant's clinical efficacy. Similar improvements in daytime functioning have been reported with lemborexant, another dual orexin receptor antagonist [23]; however, further comparative studies are needed to clarify potential differences in daytime functional effects among DORAs.

Consistency of efficacy across insomnia severity

This study suggests that daridorexant may be effective in patients with moderate to severe insomnia. An observational study conducted in Italy evaluated the efficacy of daridorexant according to insomnia severity as measured by ISI and similarly reported that daridorexant's efficacy was not influenced by baseline severity [24]. Similar findings have been reported in an observational study of lemborexant, which demonstrated significant improvements in insomnia across all severity levels (mild, moderate, and severe) [25]. These consistent findings across different DORAs suggest that efficacy independent of baseline insomnia severity, particularly in patients with severe insomnia, may be a characteristic class effect of orexin receptor antagonists. These results suggest that daridorexant may offer consistent therapeutic benefits across a spectrum of insomnia severity levels.

Efficacy in patients with suboptimal response to previous treatments

In our study, daridorexant led to notable decreases in AIS total scores even among participants who had previously received hypnotic treatments, indicating its potential utility in individuals with suboptimal responses to existing therapies. An observational study conducted in the USA reported that after more than 30 days of treatment with daridorexant, patients with an incomplete response to various prior agents experienced clinically meaningful and statistically significant improvements in ISI scores. The mean ISI in the overall cohort decreased from 18 (indicative of moderate insomnia) to below the clinical threshold for insomnia (≤11) [22]. These findings suggest that switching from conventional regimens that activate sleep-promoting pathways to alternative agents such as orexin receptor antagonists that suppress arousal-promoting pathways may offer therapeutic advantages in patients with inadequate responses to conventional hypnotics.

Effectiveness of daridorexant in patients with prior hypnotic use

A decrease in the AIS total score was also observed in the participants with prior hypnotic use in this study. Conversely, a retrospective observational study conducted in the USA reported insufficient efficacy of daridorexant in participants who switched from BZDs [22]. Another observational study demonstrated clinical improvement with daridorexant, and the proportion of patients requiring BZDs or Z-drugs decreased from 57.6% (38/66) at baseline to 13.3% (8/60) after three months of daridorexant treatment [26]. Furthermore, lemborexant has been reported to improve insomnia symptoms in patients with insufficient response to suvorexant [27]. These findings underscore the need for further evaluation of daridorexant as a therapeutic option, including switching and add-on strategies, not only for patients with inadequate response to BZDs and Z-drugs but also for those with insufficient efficacy from other DORAs. These mixed findings highlight the need for further investigation into optimal switching or combination strategies, including switch and add-on approaches, for participants with prior hypnotic medication.

Efficacy of daridorexant in patients with psychiatric comorbidities

Participants with psychiatric comorbidities also showed improvements in AIS scores. An observational study conducted at the Insomnia Clinic of the University of Pisa reported not only significant improvements in insomnia symptoms over a three-month period but also reductions in comorbid depressive symptoms following daridorexant treatment [26]. A systematic review of outcomes in individuals with insomnia and comorbid psychiatric disorders, including studies on suvorexant and lemborexant, revealed that DORAs are effective in this population [28]. These findings have prompted further clinical investigations exploring the role of DORAs in treating insomnia with co-occurring depression [29]. In the present study, daridorexant demonstrated efficacy not only in patients with comorbid depression but also in those with anxiety disorders and schizophrenia. While the majority of patients showed symptom improvement regardless of the type of comorbid psychiatric disorder, worsening of insomnia symptoms was observed in some cases. These findings highlight the importance of carefully monitoring changes in both insomnia and comorbid psychiatric symptoms during daridorexant treatment.

Safety

Regarding the safety profile of daridorexant, the primary TEAEs reported in a 52-week phase 3 study conducted in Japan were pyrexia, malaise, somnolence, headache, and nasopharyngitis [30]. In this study, malaise was the only adverse event observed, and no participants required treatment discontinuation. Furthermore, no unlisted adverse event that could pose additional risks beyond those observed in clinical trials was noted in this study, indicating that no new safety concerns emerged.

Limitations

This study has several limitations. First, as an observational, single-arm study without randomization or blinding, causal relationships cannot be definitively established, and uncontrolled confounding factors may have influenced outcomes. Second, reliance on subjective self-reported measures (AIS) without objective sleep parameters (actigraphy or polysomnography) limits assessment comprehensiveness. Third, psychiatric comorbidities in many participants may themselves influence insomnia and treatment response, acting as confounding factors that could not be adequately controlled. Fourth, exclusion of participants without week 4 assessments and those who discontinued treatment may have introduced attrition bias. Treatment selection bias also cannot be ruled out. Fifth, the small sample size precluded multivariate analyses to control for confounding factors and limited subgroup analyses. Sixth, the four-week observation period limits assessment of long-term effectiveness, safety, tolerance, and rebound effects. Finally, recruitment from three psychiatric clinics in Japan with specific exclusion criteria limits generalizability to broader populations and different clinical settings.

## Conclusions

This real-world observational study demonstrated that daridorexant effectively improved insomnia symptoms in Japanese participants, with nearly half of the participants achieving remission (AIS ≤ 5) at week 4. Notably, improvements were observed not only in sleep-related parameters but also in daytime functioning (AIS-7), addressing a key therapeutic goal in insomnia management. Importantly, therapeutic benefits were observed across participants with varying baseline insomnia severity, prior hypnotic use, and psychiatric comorbidities, populations typically excluded from clinical trials. The safety profile was favorable, with adverse events being predominantly mild and transient.

While causal inferences are limited by the observational design, these real-world findings complement existing clinical trial data and support the consideration of daridorexant as a treatment option for insomnia in routine psychiatric practice, including patients with complex clinical presentations.
